# Predictors of Cage Subsidence After Oblique Lumbar Interbody Fusion

**DOI:** 10.3390/jcm14248956

**Published:** 2025-12-18

**Authors:** Bongmo Koo, Jiwon Park, Jae-Young Hong

**Affiliations:** Department of Orthopaedic Surgery, Korea University Ansan Hospital, Korea University College of Medicine, 123 Jeokgeum-ro, Danwon-gu, Ansan 425-707, Republic of Korea; rnqhdah@gmail.com (B.K.); jwpark506@gmail.com (J.P.)

**Keywords:** spinal fusion, cage subsidence, osteoporosis, Hounsfield unit, magnetic resonance imaging

## Abstract

**Background/Objective**: Oblique lumbar interbody fusion (OLIF) achieves indirect decompression through restoration of disc height. Because maintenance of the restored disc space is essential for sustained neural decompression, solid fusion without cage subsidence is a key determinant of successful surgical outcomes. This study aimed to evaluate preoperative and intraoperative predictors of cage subsidence and radiographic fusion after OLIF. **Methods**: Seventy patients (119 levels) who underwent OLIF using a polyether–ether–ketone cage and posterior screw fixation between 2015 and 2023 were retrospectively reviewed. Preoperative bone quality was assessed using the computed tomography-based Hounsfield unit (HU) and magnetic resonance imaging-based vertebral bone quality (VBQ) score on T1-weighted images. Radiographic parameters of anterior and posterior disc height (ADH, PDH), segmental and lumbar lordotic angle (SLA, LLA), foraminal height (FH), and cage position were measured preoperatively at one-year follow-up. **Results**: Cage subsidence occurred in 21.0% of spinal levels (25/119 levels). Multivariate analysis identified these measures as independent predictors: HU (OR 1.017; *p* = 0.012), VBQ score (OR 2.716; *p* = 0.016), and PDH distraction (OR 1.418; *p* = 0.019). ROC analysis identified cutoff values of HU < 145.86 (AUC = 0.654), VBQ score > 3.30 (AUC = 0.723), and PDH distraction > 4.79 mm (AUC = 0.672). None of the evaluated factors were significantly associated with one-year radiographic fusion. **Conclusions**: Lower HU, higher VBQ score, and excessive PDH distraction are independent risk factors for cage subsidence after OLIF, although these factors do not appear to affect short-term fusion outcomes.

## 1. Introduction

Oblique lumbar interbody fusion (OLIF) is a minimally invasive technique for treating lumbar degenerative disc disease by indirectly decompressing neural elements through intervertebral disc height restoration. Unlike traditional posterior approaches, OLIF preserves posterior structures and reduces injury to paraspinal muscles [1]. Unbuckling of the ligamentum flavum contributes to central canal decompression [2], and improvements in posterior disc height and foraminal height are commonly observed postoperatively [3]. Owing to these advantages, OLIF has gained widespread use and has been shown to provide favourable clinical outcomes with decreased trauma, reduced blood loss, and faster recovery compared to conventional techniques such as posterior lumbar interbody fusion (PLIF) and transforaminal lumbar interbody fusion (TLIF) [1,4].

However, cage subsidence remains a common and significant complication. It refers to the sinking of the interbody cage into adjacent endplates, resulting in reduced disc height, loss of lordosis, recurrent foraminal stenosis, and poor outcomes including radiculopathy and mechanical back pain [5,6,7]. Reported subsidence rates range from 10% to 46.7%, highlighting the importance of identifying predictive risk factors [8,9]. In a recent systematic reviews, the median subsidence rates were reported as 17.6% for OLIF with posterior instrumentation, 21.4% for TLIF, and 15.8% for instrumented PLIF with posterior instrumentation, indicating comparable incidence across approaches despite differences in surgical corridors and cage geometry [10]. Several risk factors have been implicated, including older age, osteoporosis, endplate injury, and excessive disc height restoration during surgery [5,8]. Bone quality is critical for mechanical stability. Although T-score assessment via the DXA bone density test is a standard protocol for evaluating osteoporosis, it is not always feasible for all patients. Therefore, radiologic surrogates for bone quality have been proposed. One such surrogate is vertebral bone density assessed using the Hounsfield unit (HU) in computed tomography (CT) scans. Lower HU values, reflecting decreased vertebral strength, have been linked to a higher risk of subsidence due to inadequate load-bearing capacity [8,11]. Another surrogate is the vertebral bone quality (VBQ) score, a magnetic resonance imaging (MRI)-based measure of trabecular architecture and fat infiltration, which have been associated with osteoporosis [12,13,14]. Furthermore, Huang et al. found that a higher VBQ score significantly correlates with increased subsidence after OLIF [6]. Because preoperative imaging is routinely performed, identifying radiographic predictors of subsidence may support surgical decision-making.

Recent studies have demonstrated that cage subsidence after OLIF is also significantly influenced by cage-related and endplate-related factors. Endplate integrity, endplate morphology, and cage–endplate angular mismatch have been reported as critical biomechanical determinants of subsidence. In particular, Chen et al. showed that increased endplate injury, decreased endplate concavity, and greater cage–endplate angular mismatch were independently associated with cage subsidence [15]. In addition, comparative studies have shown that titanium cages are associated with a lower incidence of subsidence than polyetheretherketone (PEEK) cages in lateral lumbar interbody fusion, highlighting the importance of cage material in load sharing and implant stability [16].

While the association among HU, VBQ score, and subsidence has been described, few studies have evaluated their relationship to postoperative fusion. It is hypothesized that significant subsidence (>4 mm), caused by an unstable intervertebral environment, may impede fusion and increase the risk of pseudoarthrosis [5]. However, evidence confirming this link remains limited. This study aimed to investigate the relationships between HU, VBQ score, other sagittal parameters, and the occurrence of cage subsidence and radiographic fusion after OLIF. Clarifying these associations may assist surgeons in patient selection, risk stratification, and operative planning.

## 2. Methods

### 2.1. Study Design and Population

This retrospective study included 70 patients (119 operated levels) who underwent OLIF with posterior screw fixation between January 2015 and August 2023. The study was approved by the Institutional Review Board of Korea University Ansan Hospital on September 26, 2023 (IRB No. 2023AS0282). All patients presented lumbar degenerative disease, including spinal stenosis, degenerative spondylolisthesis, spondylolytic spondylolisthesis, or scoliosis, and underwent OLIF as a surgical treatment. All surgeries were performed by the same surgeon using a suitably sized 6° degree lordosis polyether–ether–ketone (PEEK) cage (Clydesdale, Medtronic Sofamor Danek, Memphis, TN, USA). Patients were included if their medical records contained preoperative lumbar CT, MRI, and radiographs, all performed within one month prior to surgery. An additional eligibility requirement was that follow-up imaging—CT and radiographs—at least one year postoperatively. Patients with a history of previous lumbar surgery, trauma, infection, or tumour or those with a metabolic disease other than osteopenia or osteoporosis were excluded. Use of anti-osteoporotic medications, including bisphosphonates, denosumab, or osteo-anabolic agents, was recorded; however, this variable was not included in the multivariate analysis due to the limited number of treated patients. Cage height was determined intraoperatively based on trial cage fitting, restoration of disc height, and resistance of the endplates. And all procedures were performed by a single surgeon using a consistent surgical strategy throughout the study period. 

### 2.2. Radiologic Evaluation

**Figure 1:**
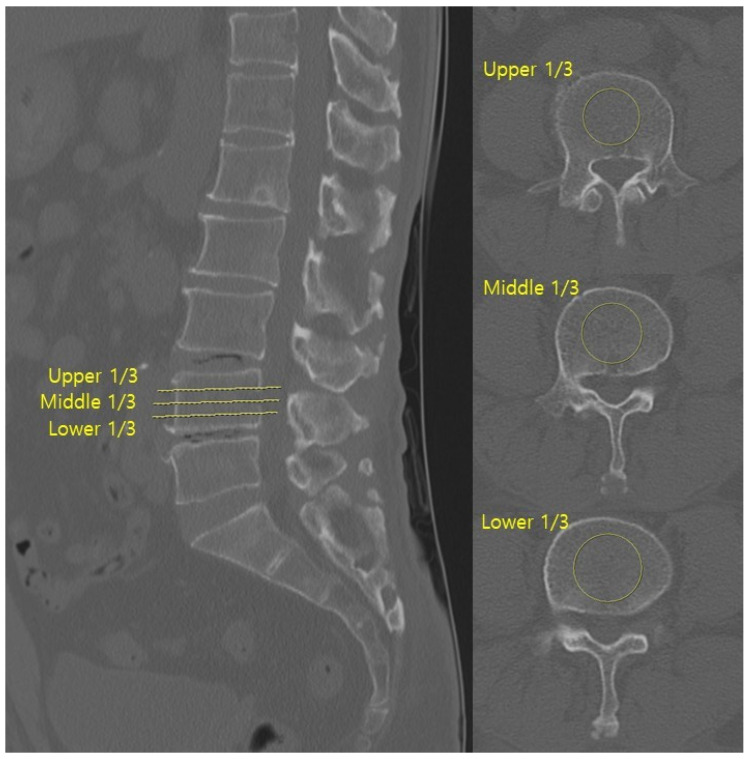
Hounsfield units were measured on axial CT images by averaging the values from three ROIs within the trabecular bone of both adjacent vertebrae at the surgical level.

HU values were measured on axial CT images at three levels (upper, middle, and lower thirds) of both the superior and inferior vertebrae body adjacent to the surgical disc space (Figure 1). Regions of interest (ROIs) were placed centrally in the trabecular bone, avoiding cortical margins and venous channels. The centre of each ROI was targeted to the central portion of the vertebral body, and the ROI area was set to include more than 50% of the vertebral body cross-sectional area while avoiding inclusion of the cortical bone.

Metal artefacts from posterior instrumentation were carefully avoided during ROI placement. All CT images were acquired with a slice thickness of 1–3 mm. The mean of three measurements for each vertebra was calculated, and the final HU value per surgical level was the average of the superior and inferior values [17]. CT scans were obtained using a multidetector CT scanner (Ingenuity, Philips Healthcare, Eindhoven, The Netherlands).

**Figure 2:**
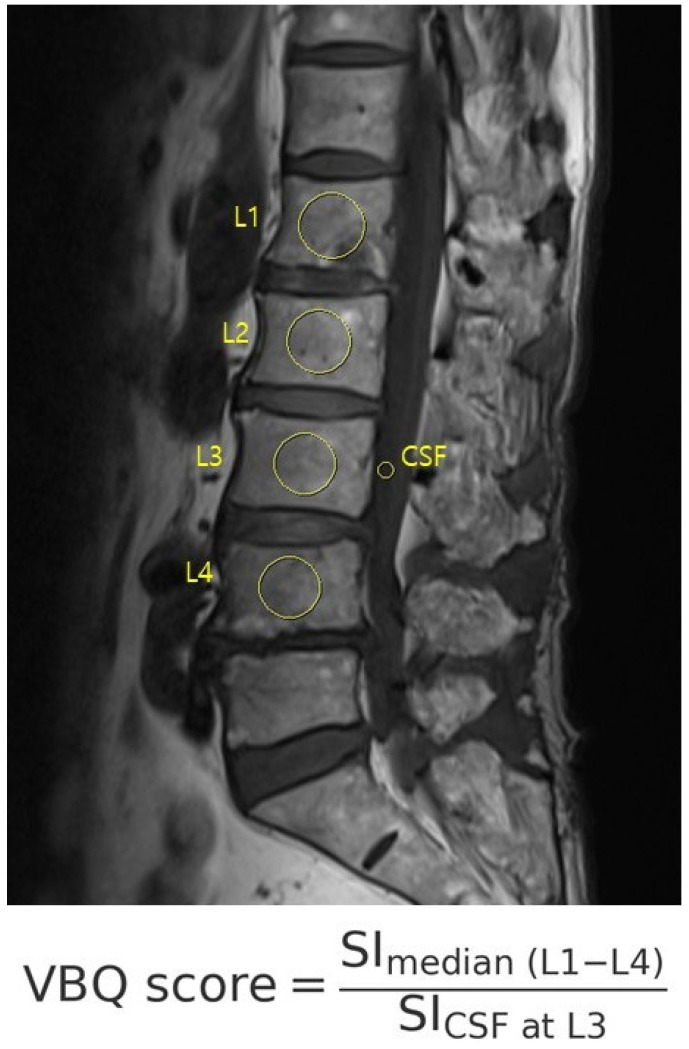
The VBQ score was calculated on T1-weighted MRI as the ratio of the vertebral body to CSF signal intensity, using manually placed ROIs from L1 to L4 and CSF at L3.

The VBQ score was calculated using non-contrast, T1-weighted MRI acquired with a 1.5 T scanner (Figure 2). ROIs were manually placed (1) within the medullary portion of L1 to L4 vertebral bodies and (2) within the cerebrospinal fluid at the L3 level. The VBQ score was defined as the ratio of the median signal intensity of the vertebral bodies to that of the cerebrospinal fluid (CSF). VBQ score = SI_ median(L1–L4)/SI_CSF at L3. Measurements were independently performed by two trained researchers who were blinded to the quantitative computed tomography (QCT) results. One investigator repeated the measurements for intra-rater reliability. The final VBQ score was the mean of four measurements [12,13,14]. MRI was performed using a 1.5-T scanner (MAGNETOM Vida, Siemens Healthineers, Erlangen, Germany).

**Figure 3:**
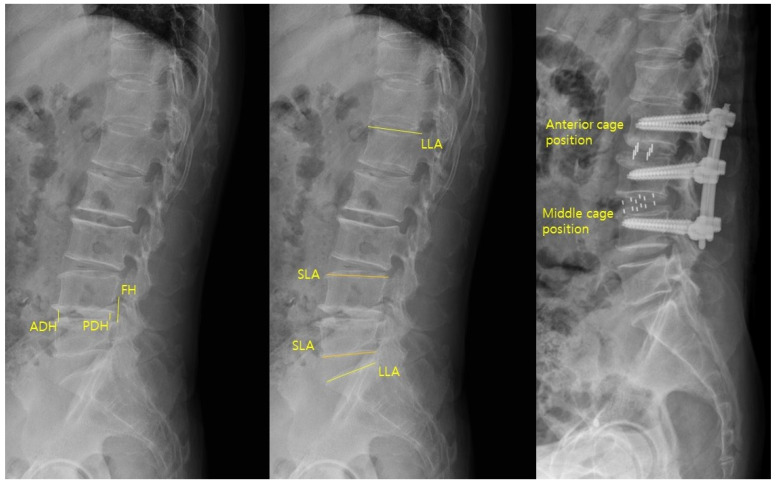
Sagittal parameters of ADH, PDH, FH, SLA, and LLA were measured on lateral radiographs, and cage position was categorized as anterior or middle based on its location on the inferior endplate.

Sagittal parameters were assessed using preoperative and postoperative lateral lumbar radiographs (Figure 3). The parameters were anterior disc height (ADH), posterior disc height (PDH), foraminal height (FH), segmental lordotic angle (SLA), and lumbar lordotic angle (LLA). ADH and PDH were defined as the vertical distances between the superior and inferior endplates at the anterior and posterior margins of the surgical level, respectively. FH was the maximum vertical distance between the inferior pedicle of the upper vertebra and the superior pedicle of the lower vertebra. SLA was the Cobb angle between the superior endplate of the upper vertebra and the inferior endplate of the lower vertebra at the surgical level. LLA was the Cobb angle from the superior endplate of L1 to the superior endplate of S1. Postoperative cage position was categorized as anterior one-third (anterior group) or middle one-third (middle group) of the inferior endplate [3]. All measurements were performed using PACS workstation software (INFINITT G3, INFINITT Health care, Seoul, Republic of Korea).

### 2.3. Cage Subsidence and Fusion Rate

The primary outcomes were cage subsidence and fusion rate at one year postoperatively. Cage subsidence was defined as a ≥2 mm decrease in posterior disc height from immediate postoperative measurements to follow-up radiographs or CT scans at the 1-year postoperative follow-up [8,9,10]. In multi-level cases, any level with subsidence was included in the subsidence group. Fusion was assessed using CT and dynamic flexion–extension radiographs at one year. Successful fusion on CT was defined as Bridwell classification grade 1 or 2, and minimal motion (<5° angular motion and <2 mm translation) on dynamic radiographs [18]. All radiologic parameters, including sagittal measurements, HU, VBQ scores, were independently evaluated by two orthopedic spine surgeons, with inter- and intraobserver reliability confirmed using intraclass correlation coefficients (ICC > 0.85 for all parameters).

### 2.4. Clinical Outcomes

Clinical outcomes were assessed using the Visual Analog Scale (VAS) score for back and leg pain at preoperative, immediate postoperative, three-month, and one-year follow-up time points.

### 2.5. Statistical Analysis

Statistical analysis was performed using SPSS software (version 20.0, IBM, Armonk, NY, USA). Continuous variables were presented as mean ± standard deviation, and categorical variables were summarized using count and percentage. Radiographic measurements were obtained by two orthopedic spine surgeons. Comparisons of continuous variables between groups with and without subsidence or successful fusion were performed using independent *t*-tests or Mann–Whitney U tests as appropriate, while categorical variables were analyzed using chi-square tests or Fisher’s exact tests. Binary logistic regression analysis was performed to identify independent predictors of cage subsidence and fusion failure at one-year follow-up. Variables significantly associated with outcomes (*p* < 0.05) in univariate analyses were included in multivariable regression models. Odds ratios (OR) and 95% confidence intervals (CI) were calculated to quantify the strength of these associations. A *p*-value less than 0.05 was considered statistically significant.

## 3. Results

A total of 70 patients (119 operated levels) met the inclusion criteria. Of these, 20 patients (28.6%) experienced cage subsidence, while 50 (71.4%) did not. As shown in Table 1, no significant differences were observed between the two groups in terms of age, sex, BMI, fusion levels, or VAS score. The mean age was 67.8 years in the non-subsidence group and 71.0 years in the subsidence group (*p* = 0.141). Single-level fusion was performed in 22 patients and multi-level fusion was conducted in 48 patients. VAS improvement was 4.18 vs. 3.25 (*p* = 0.053).

Table 2 compares the radiologic parameters by level. Of the 119 levels, 94 (79.0%) did not experienced subsidence, and 25 (21.0%) showed subsidence. Diagnoses included degenerative scoliosis (45.4%), spinal stenosis (33.6%), spondylolisthesis (10.1%), and retrolisthesis (10.9%). All cages were PEEK of 8 mm (6 levels), 10 mm (59 levels), 12 mm (51 levels), or 14 mm (3 levels). The HU values were significantly lower in the subsidence group (121.6 ± 33.3 vs. 157.7 ± 52.3; *p* = 0.006), while the VBQ scores in that group were higher (3.71 ± 0.54 vs. 3.19 ± 0.65; *p* = 0.001). Cage placement was anterior in 57 levels and middle in 62 levels; none were posterior. The average PDH distraction was significantly greater in the subsidence group (4.30 ± 1.86 mm) than in the non-subsidence group (3.07 ± 1.79 mm; *p* = 0.003). Changes in ADH, FH, LLA, and SLA showed no significant differences.

Table 3 presents the fusion outcomes. Fusion at one year was observed in 98 levels (82.4%), while. The HU and VBQ score showed no statistical association with fusion status (HU: *p* = 0.077; VBQ score: *p* = 0.544). Anterior cage placement was more common in fused levels, although it was not significant (*p* = 0.061). Changes in ADH, PDH, FH, LLA, and SLA were not significantly different between the fusion and non-fusion groups. Cage subsidence occurred in 13 of 98 fused levels (13.3%) and in 8 of 21 non-fused levels (38.1%), showing a trend toward association but without significance (*p* = 0.084).

Multivariate logistic regression identified three independent predictors of cage subsidence: lower CT-based HU, higher MRI-based VBQ score, and greater posterior disc height (PDH) distraction (Table 4). The OR for HU was 1.017 (95% CI: 1.004–1.031; *p* = 0.012), indicating that a decrease in HU was significantly associated with increased risk of subsidence. The VBQ score also showed a significant association (OR = 2.716; 95% CI: 1.208–6.104; *p* = 0.016), as did PDH distraction (OR = 1.418; 95% CI: 1.059–1.898; *p* = 0.019).

**Figure 4:**
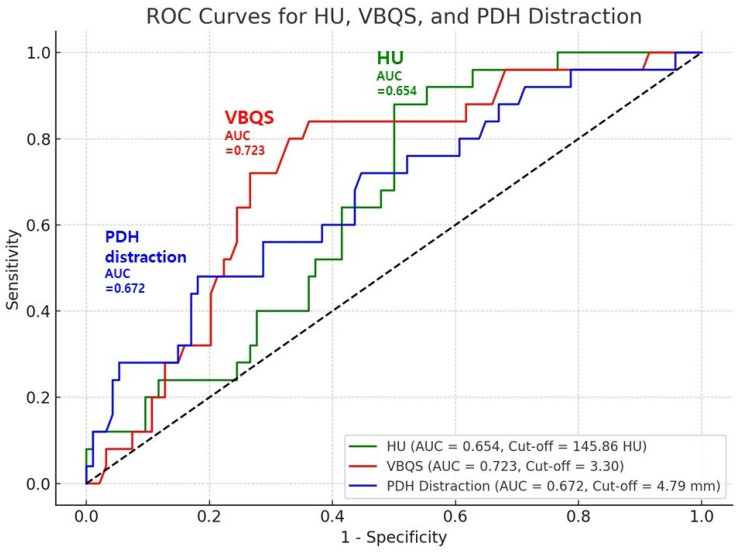
ROC analysis identified cut-off values for HU, VBQ score, and PDH distraction, supporting their predictive value for cage subsidence after OLIF.

Receiver operating characteristic (ROC) curve analysis yielded optimal cut-off values of 145.86 HU (AUC = 0.654; sensitivity = 88.0%, specificity = 50.0%), 3.30 for VBQ score (AUC = 0.723; sensitivity = 84.0%, specificity = 63.8%), and 4.79 mm for PDH distraction (AUC = 0.672; sensitivity = 72.0%, specificity = 55.3%), supporting their utility as predictive markers (Figure 4). These results suggest that poor bone quality and excessive disc distraction contribute significantly to the risk of cage subsidence after OLIF surgery.

## 4. Discussion

In this study, we identified three independent pre- or intraoperative predictors of cage subsidence following OLIF surgery: lower CT-based HU, higher MRI-based VBQ score, and greater PDH distraction. Our finding of a significant association between lower HU and increased risk of subsidence aligns with those of previous studies that highlight HU as a surrogate marker for bone mineral density and vertebral strength. Our ROC analysis yielded a cut-off value of 145.86 HU (AUC = 0.654), whereas a prior study reported a lower threshold of 120 HU with a markedly higher diagnostic accuracy (AUC = 0.906). This discrepancy may be attributed to variations in study populations or imaging protocols. Nonetheless, both findings support HU as a practical tool for preoperative risk assessment [17].

The VBQ score, a relatively novel MRI-based qualitative bone scoring system, was also significantly associated with subsidence. A higher VBQ score, reflecting reduced marrow signal intensity on T1-weighted images, likely indicates advanced trabecular bone degeneration. Our VBQ score cut-off value of 3.30 (AUC = 0.723) is comparable to previously published thresholds. Huang et al. proposed a VBQ score cut-off of 3.435 with a higher diagnostic performance (AUC = 0.839) for predicting cage subsidence after OLIF [6]. In addition, a more recent study assessed the VBQ score exclusively at the endplate level, referred to as the Endplate Bone Quality (EBQ) score, and reported that it effectively predicted cage subsidence. The proposed cut-off value in that study was 2.318 with an AUC of 0.811 [19]. Although our cut-off value demonstrates slightly lower discriminative power, it supports the utility of the VBQ score as a radiation-free adjunct in preoperative assessment of bone quality.

Owing to insurance constraints and practical limitations, DXA is not routinely performed for all surgical candidates. It should also be acknowledged that the DXA-derived T-score is substantially influenced by genetic and epigenetic factors, as well as by ethnicity and environmental background. Indeed, the incidence of osteoporotic fragility fractures shows significant geographical variation worldwide, as demonstrated by Himic et al., who reported marked regional differences in fracture risk despite comparable T-score distributions [20]. Furthermore, degenerative changes in the lumbar spine, including osteophyte formation, endplate sclerosis, and vascular calcification, have been shown to falsely elevate lumbar spine BMD on DXA, potentially leading to underestimation of true osteoporosis severity, as reported in large longitudinal studies of elderly populations [21,22].

In such cases, the HU and VBQ scores serve as valuable substitutes for assessing bone integrity. While several prior studies independently examined the HU or VBQ score in relation to spinal instrumentation failure or subsidence [6,8,11], few studies have incorporated them both in the same multivariate model. Our finding that both HU and VBQ scores are independent predictors is noteworthy, especially given that they both evaluate bone quality. This may be because HU and VBQ scores measure bone quality using different imaging principles. The HU score reflects bone mineral density through X-ray attenuation on CT, while the VBQ score assesses trabecular degeneration by comparing vertebral marrow signal intensity to CSF on T1-weighted MRI. Furthermore, statistical analysis confirmed a lack of collinearity between the two variables, justifying their simultaneous inclusion. Further research may clarify the exact relationship between these parameters.

PDH distraction was another independent predictor of subsidence. While adequate disc height restoration is necessary for effective indirect decompression, excessive distraction may compromise endplate integrity. The cut-off value of 4.79 mm (AUC = 0.672) highlights a threshold beyond which subsidence risk increases. Although the AUC was modest, the sensitivity of 72% suggests that exceeding this threshold may significantly increase the likelihood of subsidence. PDH distraction is partly iatrogenic, reflecting intraoperative cage height selection and segmental laxity, and its correlation with preoperative disc height or degeneration was not separately analyzed in this study. Previous studies have also emphasized the importance of dorsal (posterior) disc height. Iwasaki et al. reported that smaller preoperative dorsal disc height is associated with greater clinical improvement following indirect decompression via OLIF, indicating its prognostic relevance for surgical outcomes [23]. On the other hand, a recent study observed that patients in the subsidence group exhibited significantly greater disc height changes compared to those without subsidence (3.59 mm vs. 2.93 mm, *p* = 0.0238), suggesting that excessive distraction may engender a predisposition to endplate compromise [19]. To date, few studies have proposed an ideal PDH distraction threshold, further underscoring the clinical significance of our findings.

Although the ROC-derived cutoff values for HU, VBQ score, and PDH distraction were statistically significant, the corresponding AUC values indicate only moderate discriminative ability. Therefore, these thresholds should be interpreted cautiously and should not be regarded as absolute indicators for clinical decision-making. Rather, these parameters are better considered as adjunctive tools for risk stratification, to be interpreted in conjunction with other patient- and surgery-related factors, rather than as standalone diagnostic thresholds.

### Limitation of the Study and Future Research

Interestingly, while all three variables were associated with subsidence, none showed a significant correlation with one-year fusion. Prior studies have shown HU to be associated with overall osteoporosis-related complications, including pseudo-arthrosis, adjacent segment disease, and instrumentation failure [24]. However, those analyses largely aggregated multiple complications rather than isolating fusion outcomes. Notably, a separate study reported a significant association between HU and spinal fusion success. In that study, the authors evaluated 52 levels in 28 patients who underwent lateral interbody fusion and found that higher HU values were associated with increased fusion rates [25]. However, the relatively small sample size limited the generalizability of the findings. Another study reported that severe cage subsidence defined as subsidence greater than 4 mm was associated with lower fusion rates [5]. However, in our cohort, only three cases exhibited subsidence greater than 4 mm, limiting the feasibility of separately categorizing and analyzing mild versus severe subsidence. This limitation in subgroup stratification may partly explain why no significant association was observed between subsidence and fusion in our study. Early postoperative subsidence within the first 3 months was not routinely assessed in this study, as radiographic follow-up was standardized at the 1-year postoperative time point. Therefore, temporal patterns of early versus late subsidence could not be analyzed. The potential influence of perioperative anti-osteoporotic medications on cage subsidence and fusion outcomes could not be independently evaluated because of the small number of treated patients. Several considerations exist that may affect the interpretation of our findings. The HU and VBQ score are indirect markers of bone quality and are subject to variability depending on imaging protocols and measurement techniques. Although consensus is that appropriate restoration of PDH is essential for effective indirect decompression, the degree of preoperative disc space narrowing, which indicates OLIF, varies across surgeons and institutions. The required degree of distraction may differ depending on the severity and pattern of stenosis, hindering definition of a universal PDH threshold.

Although cage-related and endplate-related factors were not directly analyzed in the present study, the recent literature suggests that endplate integrity, cage–endplate angular mismatch, and cage material substantially influence the risk of subsidence after OLIF. In particular, endplate injury, decreased end-plate concavity (ECA), greater cage–endplate angular mismatch (C/EA), and the use of PEEK cages have been associated with higher subsidence rates, whereas titanium cages appear to provide more favourable load-sharing characteristics [15,16]. Therefore, our findings regarding bone quality and intraoperative distraction should be interpreted within this broader biomechanical context, and future studies integrating both radiologic bone quality indices and cage-related parameters are warranted.

Despite these limitations, our results underscore the importance of comprehensive preoperative bone quality evaluation and appropriate intraoperative disc distraction in minimizing cage subsidence risk after OLIF. Incorporating the HU and VBQ score into routine assessment may aid in risk stratification and guide surgical planning, including cage sizing, use of augmentation, or selection of osteo-anabolic agents. When planning OLIF surgery, surgeons should consider additional factors, such as patient age, degree of muscle fatty infiltration, endplate injury, and severity of preoperative stenosis.

## 5. Conclusions

Lower HU, higher VBQ score, and greater PDH distraction were independent predictors of cage subsidence after OLIF. This study is innovative in that it integrates preoperative CT- and MRI-based bone quality assessments with intraoperative mechanical factors to establish a comprehensive risk stratification model for cage subsidence. HU and VBQ score may serve as useful alternatives to T-scores for assessing bone quality when DXA is not available. Intraoperative PDH distraction should be carefully controlled. Applying these radiologic parameters during preoperative and intraoperative evaluation may help prevent subsidence and improve surgical outcomes. This study is limited by its retrospective design and relatively short 1-year follow-up period. Future studies with longer-term follow-up are warranted to further clarify the relationship between cage subsidence and long-term fusion outcomes, as well as to validate these findings in larger, multicenter cohorts.

## Figures and Tables

**Table 1:** Study group characteristics.

	Entire Group	Non-Subsidence Group	Subsidence Group	*p*-Value
Number	70	50	20	
Age (years)	68.69	67.76 ± 8.01	71.00 ± 8.72	0.141
Sex (male: female)	22:58	15:35	7:13	0.684
BMI (kg/m^2^)	25.75	25.62 ± 3.86	26.08 ± 2.62	0.630
Fusion level (N = 119)				
L2/3	27	22	5	
L3/4	47	35	12	
L4/5	45	37	8	
Fusion segment (N = 70)				0.193
Single level	22	18	4	
More than two levels	58	32	16	
Pre-VAS		7.38	7.05	
Post-VAS		3.20	3.80	
VAS change		4.18 ± 1.92	3.25 ± 1.55	0.059

Normality of continuous variables was assessed using the Shapiro–Wilk test. VAS: Visual analog scale.

**Table 2:** Non-subsidence and subsidence level comparison.

	All Levels	Non-Subsidence Levels	Subsidence Levels	*p*-Value
Number	119	94 (79.00%)	25 (21.00%)	
Segmental diagnosis				0.314
Spondylolisthesis	12 (10.1%)	11 (91.7%)	1 (8.3%)	
Retrolisthesis	13 (10.9%)	10 (76.9%)	3 (23.1%)	
Scoliosis	54 (45.4%)	39 (72.2%)	15 (27.8%)	
DDD	40 (33.6%)	34 (85.0%)	6 (15.0%)	
CT_HU (HU)	150.11	157.69 ± 52.26	121.59 ± 33.28	0.006
MRI_VBQ score	3.30	3.19 ± 0.65	3.71 ± 0.54	0.001
Cage height (mm)				0.793
8		4 (66.7%)	2 (33.3%)	
10		48 (81.4%)	11 (18.6%)	
12		40 (78.4%)	11 (21.6%)	
14		2 (66.7%)	1 (33.3%)	
Cage position (anterior: middle)		48:46	9:16	0.180
Cage-subsidence (mm)	0.87	0.35 ± 1.01	2.82 ± 0.81	0.00
ADH distraction (mm)		5.55 ± 2.93	5.99 ± 3.33	0.549
PDH distraction (mm)	3.33	3.071 ± 1.79	4.30 ± 1.86	0.003
FH change (mm)		3.89 ± 3.30	4.77 ± 2.28	0.213
LLA change (°)		2.18 ± 9.74	3.39 ± 7.16	0.564
SLA change (°)		1.97 ± 6.52	0.74 ± 5.25	0.387
Fusion	98:21	81:13	17:8	0.084

DDD: Degenerative disc disease with normal alignment; HU: Hounsfield unit; VBQ score: Vertebral bone quality score; ADH: Anterior disc height; PDH: Posterior disc height; FH: Foraminal height; LLA: Lumbar lordotic angle; SLA: Segmental lordotic angle.

**Table 3:** Fusion and non-fusion level comparison.

	All Levels	Fusion Levels	Non-Fusion Levels	*p*-Value
Number	119	98	21	
CT_HU		156.10 ± 51.46	122.19 ± 44.21	0.077
MRI_VBQ score		3.28 ± 0.71	3.38 ± 0.49	0.544
Cage position (anterior: middle)	57:62	51:47	6:15	0.061
ADH distraction (mm)		5.46 ± 3.06	6.63 ± 2.72	0.107
PDH distraction (mm)		3.28 ± 1.86	3.61 ± 1.76	0.364
FH change (mm)		3.83 ± 3.04	5.22 ± 3.41	0.077
LLA change (°)		2.14 ± 9.23	3.83 ± 9.41	0.449
SLA change (°)		1.50 ± 6.06	2.73 ± 7.54	0.415
Cage subsidence		81:17	13:8	0.084

HU: Hounsfield unit; VBQ score: Vertebral bone quality score; ADH: Anterior disc height; PDH: Posterior disc height; FH: Foraminal height; LLA: Lumbar lordotic angle; SLA: Segmental lordotic angle

**Table 4:** Multivariable logistic regression analysis predictors for subsidence.

	OR	CI	*p*-Value
CT_HU	1.017	1.004–1.031	0.012
MRI_VBQ score	2.716	1.208–6.104	0.016
PDH distraction (mm)	1.418	1.059–1.898	0.019

HU: Hounsfield unit; VBQ score: Vertebral bone quality score; PDH: Posterior disc height.

## Data Availability

The datasets generated and/or analyzed during the current study are available from the corresponding author on reasonable request.
